# Effects of Freeze–Thaw Thermal Cycles on the Mechanical Degradation of the Gas Diffusion Layer in Polymer Electrolyte Membrane Fuel Cells

**DOI:** 10.3390/polym11030428

**Published:** 2019-03-06

**Authors:** Yanqin Chen, Chao Jiang, Chongdu Cho

**Affiliations:** Department of Mechanical Engineering, Inha University, Incheon 22212, Korea; chenyanqin@inha.edu (Y.C.); chiaojiang@inha.edu (C.J.)

**Keywords:** gas diffusion layers, polymer electrolyte membrane fuel cell, repeated freeze–thaw thermal cycles, three-dimensional mechanical degradation, thermal failure, scanning electron microscope images

## Abstract

In this paper, the mechanical degradation of a commercial gas diffusion layer subjected to repeated freeze–thaw thermal cycles is studied. In a fuel cell, the mechanical assembly state directly affects the performance of polymer electrolyte membrane fuel cells. Particularly, the gas diffusion layer repeatedly withstands the complex heat and humidity environmental conditions in which the temperature and humidity are always greatly changed. Studying the three-dimensional mechanical degradation of gas diffusion layers due to orthotropic properties is very useful in extending the lifetime and durability of fuel cells. To investigate this, we first established the standard freeze–thaw thermal cycle and studied the gas diffusion layer’s mechanical degradation performance with up to 400 repeated freeze–thaw thermal cycles. Furthermore, different types of failure in the gas diffusion layer caused by the repeated thermal aging treatment were observed using a scanning electron microscope, to explain the change in the mechanical deterioration. As a result, the different thermal failure plays different roles in the explanation of the gas diffusion layer’s mechanical degradation under different thermal cycles. In particular, the thermal failure that resulted from the first 100 thermal cycles has the greatest effect on the compressive and tensile performance, compared to the shear behavior.

## 1. Introduction

**Figure 1:**
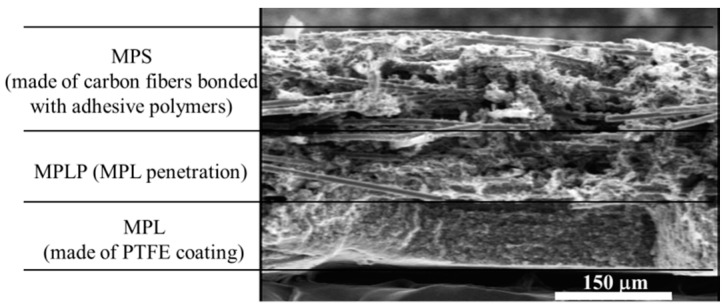
Layer partition of a gas diffusion layer (GDL).

Gas diffusion layers (GDLs) are commonly composed of a substrate (made of carbon fibers, carbon felt, carbon cloth, or metal foam), a microporous layer (MPL, usually made of polytetrafluoroethylene, PTFE), and a penetration layer between them [1,2]. Moreover, GDLs are filled with resins [3,4] among the three layers. According to our observation of a pristine commercial GDL by Scanning Electron Microscopy (Hitachi SU-8010, Tokyo, Japan), the structure of the GDL can be clearly seen in Figure 1. In polymer electrolyte membrane fuel cells (PEMFCs), GDLs are placed between the bipolar plate (BPP) with gas channels and the catalyst layer (CL) under a certain clamping load using bolts. During the operation of PEMFCs, hydrogen and oxygen are injected into the anode and cathode through the gas channels; a redox reaction then occurs in the CL, and then water, as well as heat, are produced and expelled through the porous GDLs [5]. Thus, GDLs not only suffer from severe humidity but also go through variable temperature environmental conditions. In general, PEMFCs operate within a temperature range of −20 to 120 °C and at a relative humidity (RH) within 0%–100% [6,7,8]. These intricate humidity and temperature conditions have direct effects on the mechanical performance of the GDLs, which also influences the performance of PEMFCs.

Mostly, the degradation of GDLs can be classified into mechanical degradation (including compression force effect, freeze–thaw thermal cycle effect, dissolution, and erosion effect) and physicochemical degradation (including chemical dissolution effect, carbon corrosion effect, and electrochemical degradation) [1,3]. Han et al. [9], Lee et al. [10], and Yan et al. [11] only investigated the freezing effects on the physical properties of GDLs, such as polarization curves, voltage and current performance, high-frequency resistance, thickness, surface morphology, pore size distribution, gas permeability, and contact angle of the surface. Although the effects of freeze–thaw thermal cycles on GDL degradation have been studied [3,12,13,14], the majority merely focused on the GDLs’ physical degradation performance. Very few papers have investigated the effects of repeated freeze–thaw thermal cycles on the GDLs’ mechanical performance response. In the case of mechanical failure or deformation existing in GDLs, PEMFCs’ performance, such as the voltage vs. current density curves, electrical conductivity, gas transfer efficiency, water management, and thermal conductivity would be directly affected. The mechanical aging treatment of GDLs could result in decreased cell voltage [15], poor polarization and power density curves [16], especially at high current density. An understanding of physical degradation is not sufficient, as mechanical degradation is also significant. In fact, GDLs in PEMFCs withstand intricate loads between BPPs with flow channels and CLs. Not only compressive pressure, but also tensile as well as shear loads are present in GDLs. Thus, it is more convincing to research the GDLs’ three-dimensional (3D) mechanical behavior due to orthotropic properties [17].

In this research, the GDL’s mechanical degradation is investigated through a series of repeated freeze–thaw thermal cycles. In order to make the GDL totally frozen and thawed, the freeze–thaw thermal cycle was built based on the real operative temperature and humidity conditions in fuel cells. Considering the GDLs’ orthotropic properties and real configurations in PEMFCs, the mechanical tests, such as compressive tests in the thickness direction, in-plane tension tests, and shear tests, were performed to investigate the 3D mechanical degradation behavior. In addition, failure in the GDL resulting from the repeated thermal aging treatment was observed through SEM images, to analyze changes in its mechanical degradation.

## 2. Experimental Details

### 2.1. Experimental Procedures

**Figure 2:**
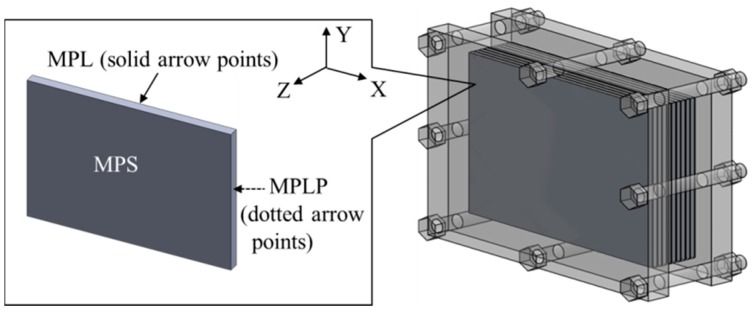
Representative image of the clamped GDLs.

In this study, a commercial GDL (JNT 30-A1, JNT Group Company, Hwasung, Korea) was employed as the sample. The original size of the commercial GDL is 300 mm × 210 mm × (320 ± 20) μm (X × Y × Z; where X is the longitudinal direction, Y is the transverse direction, and Z is the thickness direction). Before the GDL experienced repeated freeze–thaw thermal cycles, each sheet of GDL was clamped between plates by bolts under a certain load, to imitate the assembly condition in a fuel cell unit. The representative image of the clamped GDLs is shown in Figure 2.

**Figure 3:**
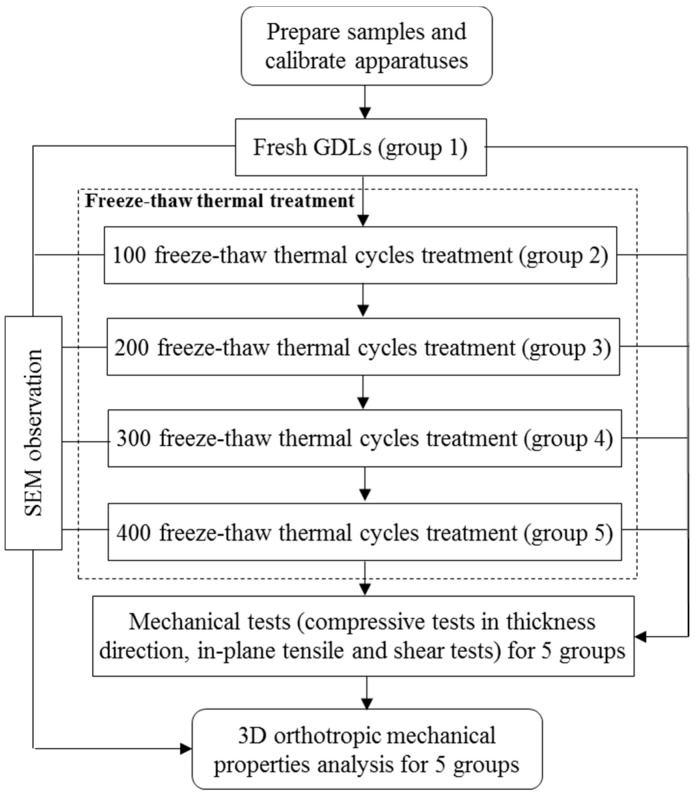
Flow chart of the experimental procedures.

A temperature and humidity control chamber TEMI 300 (JEIO TECH, Seoul, Korea) and a Material Tester BS-205 (C & FO Engineering, Osan, Korea) were respectively used to subject samples to the freeze–thaw thermal cycle aging treatment and mechanical tests. In this research, the entire thermal aging time was determined to be 1200 h. After certain freeze–thaw thermal cycles, some aging GDLs were taken out of the chamber and disassembled in an orderly manner. Then, samples tailed from the sheets of the pristine and aging GDLs were divided into five groups corresponding to different aging thermal cycles for mechanical tests, so as to observe how the GDL’s 3D mechanical degradation behaved as the number of freeze–thaw thermal cycles increased. For each group, more than five specimens captured from different locations of the pristine and aging sheet of GDLs were employed for the SEM observation. Representative images subject to different thermal aging treatments were used for the microstructure analysis. Detailed experimental procedures are illustrated in Figure 3.

### 2.2. Freeze–Thaw Thermal Cycle

**Figure 4:**
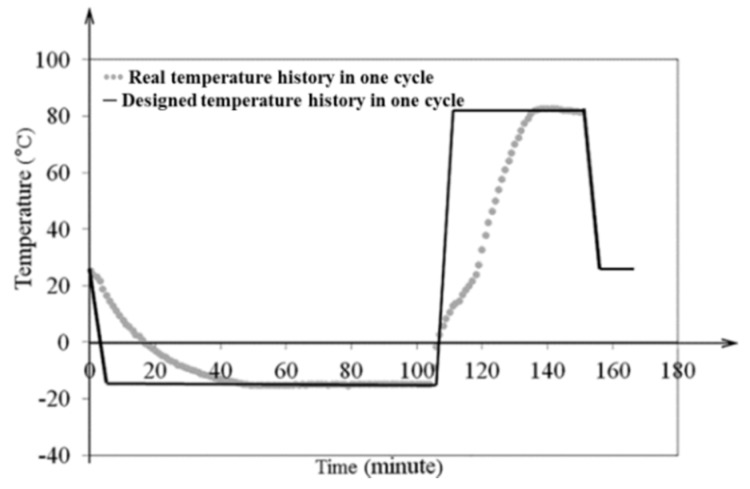
One complete freeze–thaw thermal cycle.

The freeze–thaw thermal cycle was built to investigate the GDL’s mechanical response to the effects of water frozen and thaw. Thus, samples should be completely frozen in the frozen stage, and completely thawed in the thawed stage. In fact, the microstructural breakage and deformation of most materials generally happens during freezing and thawing, due to the temperature and humidity dramatically changing. However, the maintenance stage of temperature and humidity after the GDL is completely frozen or thawed is not necessary. Considering the working temperature of most fuel cells within −20 to 120 °C, a temperature range of −15 to 80 °C was set. As for the operative humidity, the RH in fuel cells is generally between 0% and 100%. Since the relationship between temperature and RH is complex, in this research an extreme condition of 100% RH was employed for all temperature ranges. After attempting a series of freeze–thaw thermal cycles to observe the GDL’s freeze and thaw conditions, one complete freeze–thaw thermal cycle was finally established. The determined thermal cycle could ensure the GDL was completely frozen and thawed, as shown in Figure 4.

### 2.3. Mechanical Tests

Samples were tailored from the pristine and aging sheets of the commercial GDL. In a strict sense, the 3D mechanical behavior of GDLs, brittle orthotropic polymer composites in PEMFCs, should be tested. Considering GDL assembly configuration in fuel cells, mechanical tests, such as compressive tests in the thickness direction (Z direction), in-plane tensile (in X and Y directions), and shear tests (in ZX and ZY planes), were performed using a Material Tester BS-205. More detailed test methods can be found in [18,19,20,21]. Furthermore, five specimens were conducted for each test, and the average experimental data were employed to state the GDL’s mechanical degradation performance.

## 3. Results and Discussion

### 3.1. GDL’s Compressive Degradation Performance

**Figure 5:**
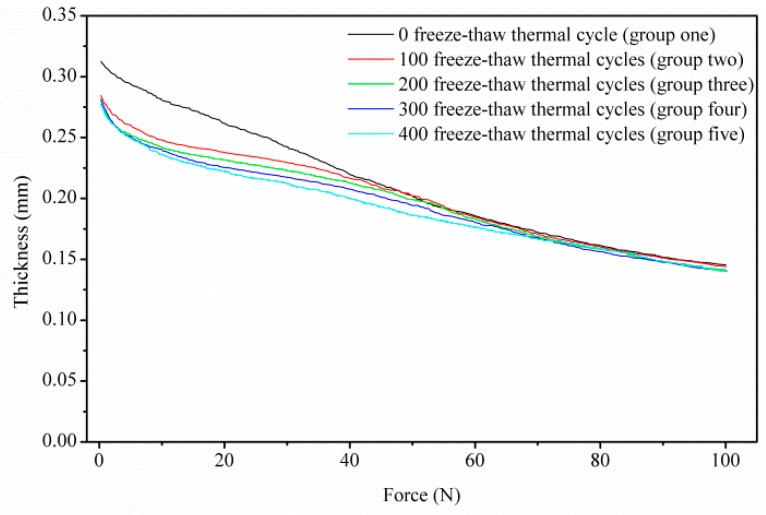
GDL’s compressive behavior through a series of freeze–thaw thermal cycles.

Figure 5 shows the average tested force–thickness curves (with the max experimental error 6.5%) through different freeze–thaw thermal cycles; the curves could be divided into two stages. Notably, at the beginning of the curves, the pristine GDL (group 1) shows distinct compressive behavior from the aging GDL (group 2 to group 5). In particular, the thickness of the aging GDL is thinner than that of the pristine GDL at free force (0 N). In fact, the assembly force acting on GDLs [22,23,24] and frost-heaving force [25,26,27] produced and maintained during the freeze–thaw thermal cycles cause the breakage of carbon fibers in the substrate, which accounts for the distinction in the first stage. Among the aging groups, compressive behavior does not display such a distinct difference. Thus, the first 100 thermal cycles of aging treatment make a significant contribution to the GDL’s compressive behavior response, with subsequent thermal cycles showing some gradual influence.

**Figure 6:**
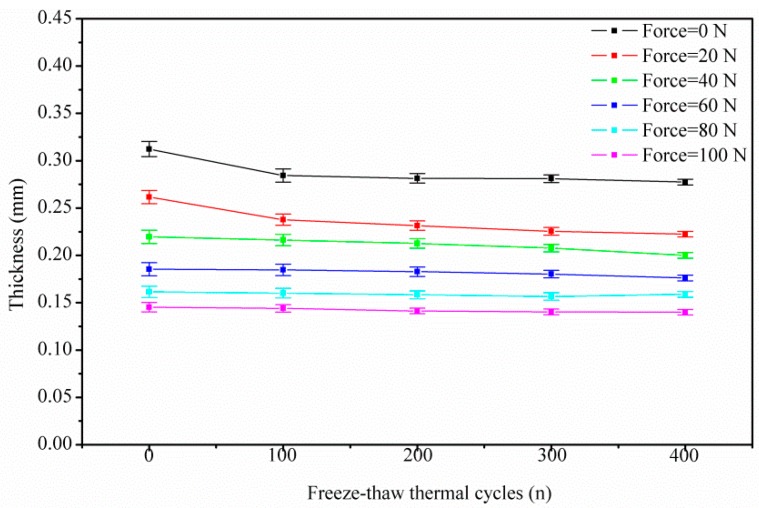
GDL’s compressive degradation under a series of freeze–thaw thermal cycles.

In cases of compressive force beyond 60 N (3 MPa, with the specimen square of 20 mm^2^ in this research), the effects of clamping and frost-heaving force on the GDL diminish. In other words, as shown in the second stage of Figure 5, as well as Figure 6, all of the five groups—regardless of whether aging or pristine GDL—show the coincident compressive response beyond 60 N. It indicates that a compressive force over 60 N could ensure that GDL is compacted to a stable state, to some extent. A threshold pressure of 3 MPa is related to the breakage and reorganization of carbon fibers. A similar report [28], stated that different GDL types have a meeting point around 3 MPa in the stress–density curves, despite the fact that the two GDLs have different area densities. Thus, once the load of 3 MPa is reached, the compressive force governs, compared to the clamping and frost-heaving force.

**Figure 7:**
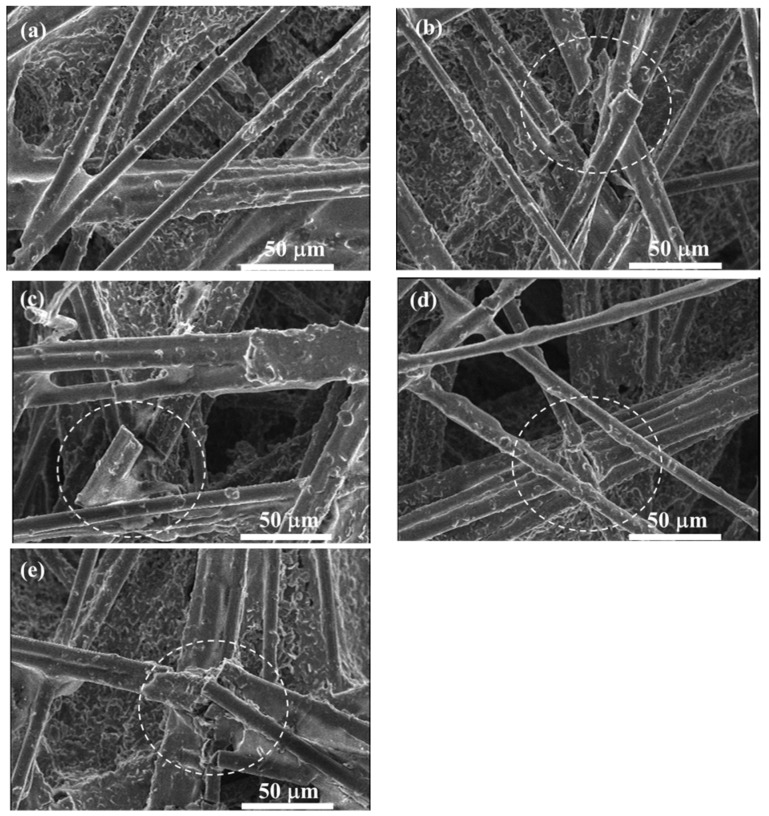
SEM images of the GDL in the substrate under a series of freeze–thaw thermal cycles: (**a**) 0 cycle; (**b**) 100 cycles; (**c**) 200 cycles; (**d**) 300 cycles; (**e**) 400 cycles.

In addition, representative SEM images (as shown in Figure 7) captured from the pristine and the aging GDLs under the same observation scale (50 μm) provide a better understanding of the microstructure transformation. Obviously, after repeated thermal aging treatment as shown in Figure 7b–e, some regions (the white dotted circle) marked with the breakage of carbon fibers are clearly observed. However, these breakages are not found in the pristine GDL in Figure 7a. The described fracture of the carbon fibers reveals the difference in force–thickness curves in the first segment in Figure 5 at the free force. Thereby, it confirms that the assembly force acting on GDLs and the frost-heaving force produced by repeated thermal aging treatment could result in the breakage of carbon fibers, and this breakage then influences the GDL’s compressive degradation response.

### 3.2. GDL’s In-Plane Tensile Degradation Performance

**Figure 8:**
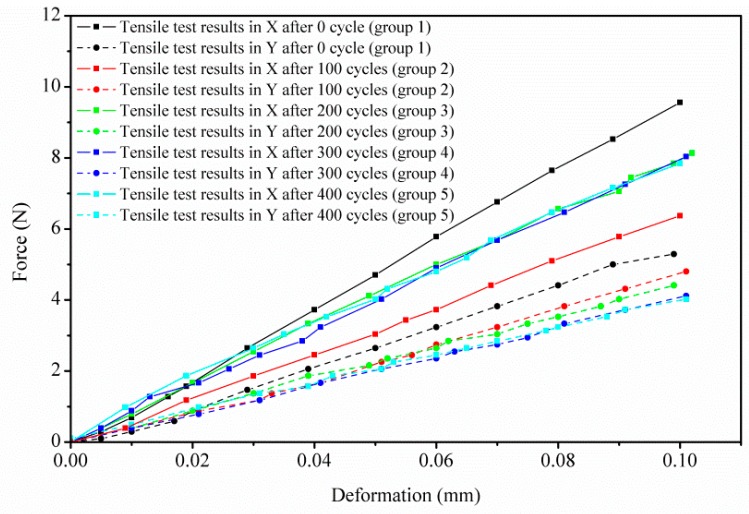
GDL’s in-plane tensile behavior through a series of freeze–thaw thermal cycles.

Figure 8 depicts the tested in-plane (X and Y directions) force versus deformation curves of the GDL experiencing different freeze–thaw thermal cycles. The slopes of the in-plane deformation–force curves (tensile stiffness, N/mm) for all the groups are almost constant. However, the tensile stiffness in the X and Y directions of the GDL is quite different, which indicates the in-plane tensile performance is anisotropic, mainly due to the carbon fiber alignments; similar results can be found in [29,30].

**Figure 9:**
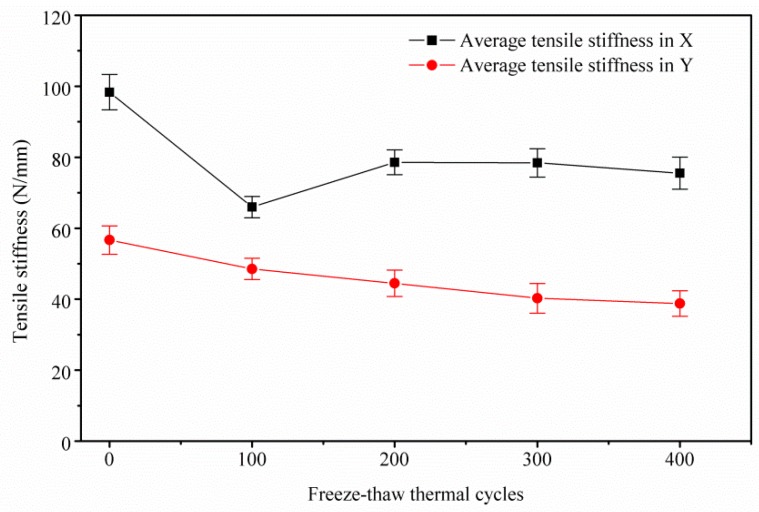
GDL’s in-plane tensile degradation under a series of freeze–thaw thermal cycles.

In Figure 9, it can be clearly observed that tensile stiffness in the X direction decreases rapidly until the first 100 freeze–thaw thermal cycles. Interestingly, tensile stiffness in the X direction recovers somewhat and then, monotonically, it almost keeps constant. However, tensile stiffness in the Y direction decreases slightly with the increase of freeze–thaw thermal cycles.

**Figure 10:**
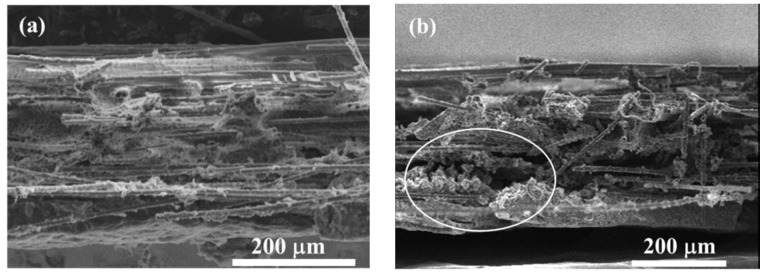

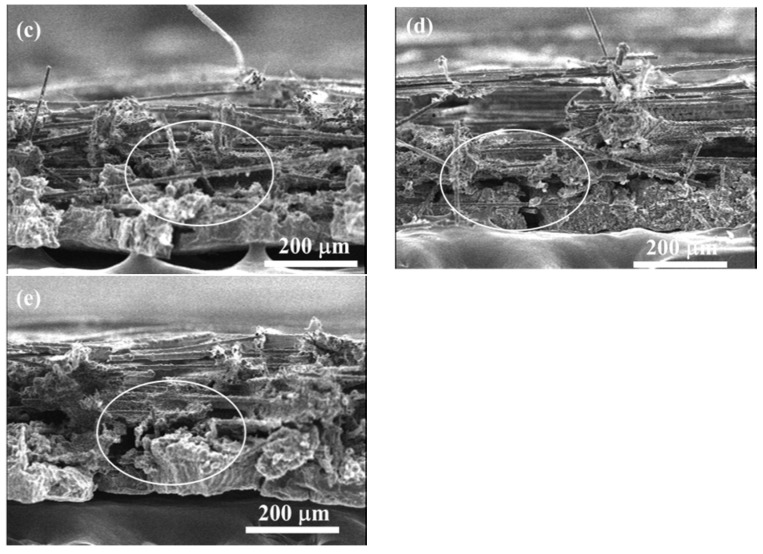
The cross-section SEM images of the GDL under a series of freeze–thaw thermal cycles: (**a**) 0 cycle; (**b**) 100 cycles; (**c**) 200 cycles; (**d**) 300 cycles; (**e**) 400 cycles.

**Figure 11:**
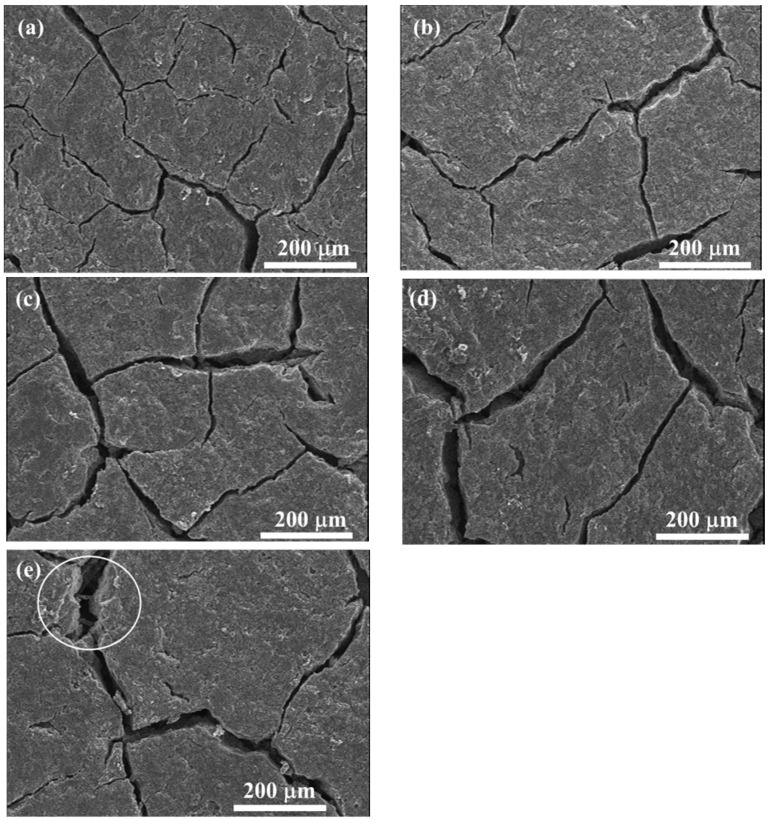
SEM images of the GDL in MPL under a series of freeze–thaw thermal cycles: (**a**) 0 cycle; (**b**) 100 cycles; (**c**) 200 cycles; (**d**) 300 cycles; (**e**) 400 cycles.

As previously described, the GDL is generally composed of a substrate, an MPL, and a penetration layer between them. In the substrate, carbon fibers are bonded together by adhesive polymers which are sensitive to temperature and humidity. After several repeated thermal aging treatment, the adhesive failure of bonded carbon fibers occurs. Moreover, the breakage of carbon fibers in the substrate can be clearly observed in Figure 7b–e. Subsequently, the carbon fiber’s breakage and the adhesive failure of bonded fibers result in the delamination and interlaminar damage issues [1,31,32] in the penetration layer, as shown in the white circles marked in Figure 10b–e. Additionally, the cracks in MPL are aggravated, which can be observed through the SEM images in Figure 11. In the pristine GDL, some tiny cracks, as shown in Figure 11a, exist because of the manufacturing process. With the increase of thermal cycles, these cracks become larger and larger due to the thermal aging effects on the PTFE coating (thermoplastic polymer, which is sensitive to temperature and humidity [33]). As a result, some regions in MPL completely crack, especially after 400 cycles, as seen in the white circle region in Figure 11e, where the carbon fibers in the substrate can be directly observed through the crack.

**Figure 12:**
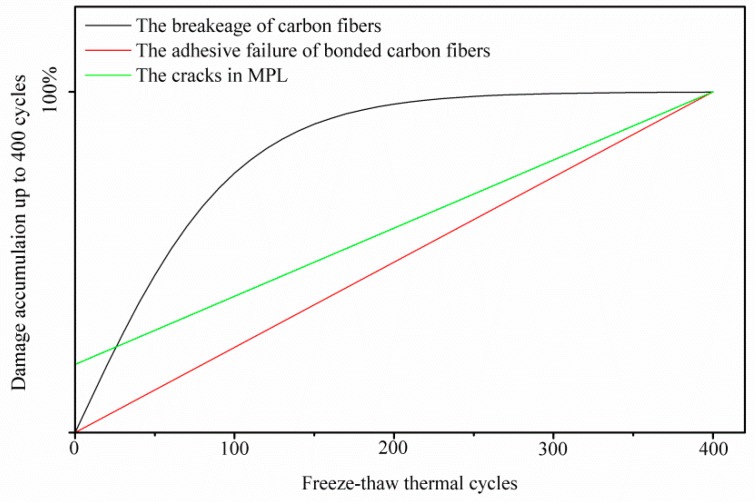
Schematic showing the dependence of damage accumulation on freeze–thaw thermal cycles, up to 400 thermal cycles.

All of the three abovementioned kinds of failure, due to the repeated thermal aging treatment, account for the GDL’s tensile degradation performance. In fact, the three kinds of failure play different roles in different aging stages. In the first 100 thermal cycles, the breakage of carbon fibers caused by the assembly force and the frost-heaving force dominates, as presented in Section 3.1. Then, the breakage of carbon fibers proportionally affects the GDL’s mechanical degradation. Differently from the response of the breakage of carbon fibers, the local adhesive failure of bonded carbon fibers and the cracks in MPL are observed as having proportional effects on the mechanical degradation all through the thermal cycles. However, the cracks in MPL seem to show a weak effect on GDL’s mechanical degradation, compared to the impact of carbon fibers damage. This is because of the fact that PTFE in MPL normally has a storage modulus of about 1 GPa [33]. The response of the cracks in MPL on the degradation of GDL is isotropic. The carbon fiber alignments in the GDL are quite different in X and Y directions. Generally, the carbon fibers have a longitudinal modulus of 225 GPa and transverse Young’s modulus of 15 GPa [34]. Thus, the in-plane tensile characteristics of the GDLs behave in an anisotropic way, and it can be easily imaged that the breakage of carbon fibers and the local adhesive failure of bonded carbon fibers should be anisotropic in X and Y directions. According to our investigation, the breakage of carbon fibers affects the tensile degradation more seriously in the X direction than that in the Y direction. The local adhesive failure of bonded carbon fibers also greatly influences tensile degradation in the X direction, compared to that in the Y direction. All three kinds of thermal failure in the GDL above are very difficult to quantify through experiments. Here, a schematic of the dependence of the damage accumulation in the GDL on freeze–thaw thermal cycles is given in Figure 12, which is responsible for the GDL’s tensile degradation performance in Figure 9.

### 3.3. GDL’s Shear Degradation Performance

**Figure 13:**
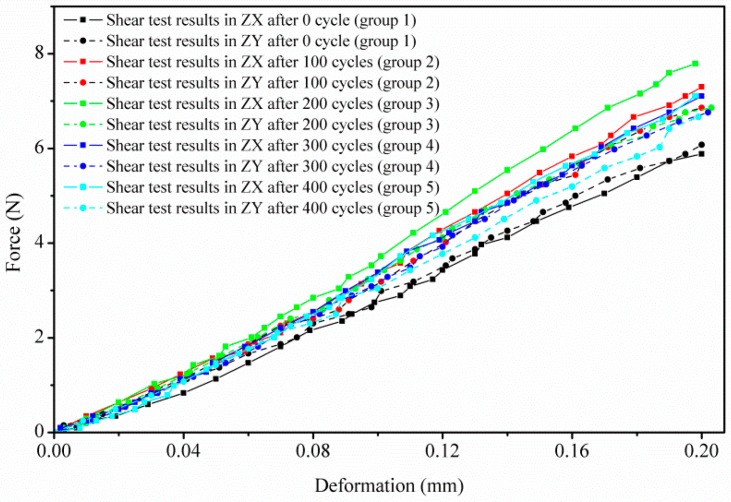
GDL’s shear behavior in ZX and ZY planes under a series of freeze–thaw thermal cycles.

**Figure 14:**
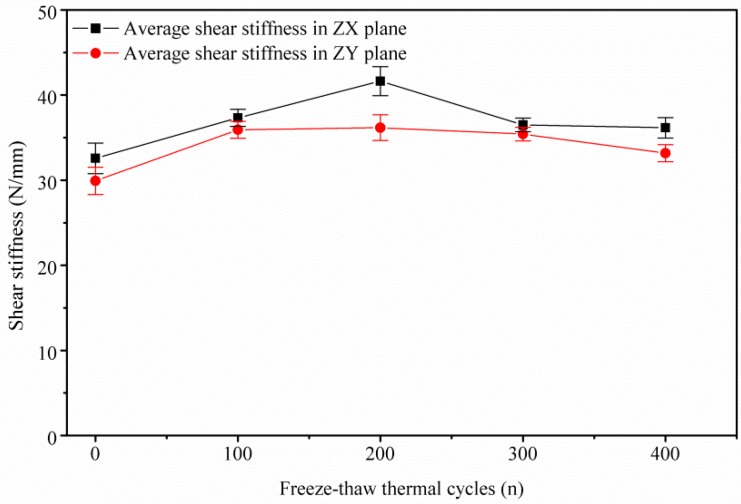
GDL’s shear degradation in ZX and ZY planes under different freeze–thaw thermal cycles.

Figure 13 shows the GDL’s shear degradation response in both ZX and ZY planes. Similar to the tensile degradation behavior, the slopes of the deformation–force curves (shear stiffness, N/mm) are also constant. Related studies about the shear modulus of GDLs were reported in [21], where the shear moduli are determined as constants through a punch-and-die type of shear test. In this research, a similar shear behavior response is obtained, as described in Figure 13 and Figure 14. Experimental shear stiffness in the ZX plane is a little larger than that in the ZY plane. Roughly, under the same plane, shear stiffness (within 32–41 N/mm in ZX or within 31–35 N/mm in ZY) almost remains stable no matter how many freeze–thaw thermal cycles, except the stiffness in the ZX plane after 200 thermal cycles. Perhaps the rebound point in shear stiffness in the ZX plane under 200 thermal cycles is related to the densification of the carbon fiber network [35,36] because of the combined action of carbon fibers’ alignments, clamping load, frost-heaving force, and thermal effects on the GDL.

In fact, in this research, the shear failure of the GDL is achieved through squeeze action by controlling the movement of tool heads. All the above mentioned failures caused by repeated thermal aging treatment, such as the fracture of carbon fibers and the adhesive bonded connection failure in the substrate, the delamination and interlaminar damage in the penetration layer, and the cracks of PTFE coating in MPL, contribute very little to the GDL’s shear degradation behavior. This also means that the freeze–thaw thermal cycles rarely influence the GDL’s shear performance.

## 4. Conclusions

This study presented the mechanical degradation behavior of a commercial GDL under several freeze–thaw thermal cycles. Based on the GDL’s orthotropic properties and the configuration in PEMFCs, the 3D mechanical performance is quantified through compressive, tensile, and shear tests. In addition, the failure in the GDL caused by the repeated thermal aging treatment, such as the breakage of carbon fibers, the adhesive failure of bonded fibers, and the cracks in MPL, are observed by SEM images. Furthermore, the thermal failure accounts for the GDL’s 3D mechanical degradation performance, to some extent. The results are summarized as follows:

- For the compressive performance, the initial phase of the repeated thermal treatment has more effects, due to the breakage of carbon fibers in the substrate caused by the assembly force and frost-heaving force. In the case of compressive loads beyond 60 N (or 3 MPa), the effects of further thermal cycles diminish and the GDL’s compressive behavior could remain stable.
- For the in-plane tensile performance, the GDL shows anisotropic properties. The repeated freeze–thaw thermal aging treatment greatly affects the GDL’s in-plane tensile behavior. All three kinds of thermal failure, that play different roles in different thermal stages, contribute to the GDL’s anisotropic tensile degradation.
- For the shear performance, although there are slight fluctuations in the shear stiffness, it could be roughly assumed that the freeze–thaw thermal cycles affect the GDL’s shear performance negligibly, according to the experimental data. As for the rebounded point, this might be due to densification of the carbon fiber network.
